# Clinical utility of liquid biopsy for EGFR driver, T790M mutation and EGFR amplification in plasma in patients with acquired resistance to afatinib

**DOI:** 10.1186/s12885-020-07777-2

**Published:** 2021-01-12

**Authors:** Yuko Oya, Tatsuya Yoshida, Kazuhiro Asada, Tetsuya Oguri, Naoki Inui, Sayako Morikawa, Kentaro Ito, Tomoki Kimura, Eiji Kunii, Takashi Matsui, Akihito Kubo, Tatsuo Kato, Takashi Abe, Takeshi Tsuda, Toyoaki Hida

**Affiliations:** 1 Department of Thoracic Oncology, Aichi Cancer Center Hospital, 1-1 Kanokoden, Chikusa-ku, Nagoya, Aichi 464-8681 Japan; 2 Current Address: Department of Thoracic Oncology, National Cancer Center Hospital, 5-1-1 Tsukiji, Chuo-ku, Tokyo, 104-0045 Japan; 3 Department of Respiratory Medicine, Shizuoka General Hospital, Shizuoka, Japan; 4 Department of Respiratory Medicine, Allergy and Clinical Immunology, Nagoya City University, Nagoya, Japan; 5 Second Division, Department of Internal Medicine, Hamamatsu University School of Medicine, Hamamatsu, Japan; 6 Department of Respiratory Medicine, Fujita Health University School of Medicine, Toyoake, Japan; 7 Respiratory Center, Matsusaka Municipal Hospital, Matsusaka, Japan; 8 Department of Respiratory Medicine and Allergy, Tosei General Hospital, Seto, Japan; 9 Department of Respiratory Medicine, Nagoya City West Medical Center, Nagoya, Japan; 10 Department of Respiratory Medicine, Seirei Mikatahara General Hospital, Hamamatsu, Japan; 11 Division of Respiratory Medicine and Allergology, Aichi Medical University School of Medicine, Nagakute, Japan; 12 Department of Respiratory Medicine, National Hospital Organization Nagara Medical Center, Gifu, Japan; 13 Department of Respiratory Medicine, Ogaki Municipal Hospital, Ogaki, Japan; 14 Department of Respiratory Medicine, Toyama Prefectural Central Hospital, Toyama, Japan

**Keywords:** Epidermal growth factor receptor (EGFR), EGFR-tyrosine kinase inhibitor, Non-small cell lung cancer (NSCLC), Afatinib

## Abstract

**Background:**

Cell-free DNA (cfDNA) genotyping in plasma using the cobas EGFR Mutation Test v2 (cobas) is the first liquid biopsy as a companion diagnosis to identify the EGFR T790M mutation (T790M) after the failure of treatment of EGFR-tyrosine kinase inhibitors (TKIs) (1st generation, gefitinib [G] and erlotinib [E] and 2nd generation, afatinib [A]). This study investigated the clinical utility of a liquid biopsy for patients who acquired resistance to afatinib.

**Methods:**

We prospectively collected plasma from 51 patients who had acquired resistance to afatinib between April 2015 and November 2016 to evaluate the frequency of T790M by cobas and digital droplet PCR (UMIN000025112). Additionally, we retrospectively reviewed 38 patients who tested by cobas in plasma after G/E failure to compare for T790M detection between A and with G/E.

**Results:**

The detection rate of EGFR-driver and T790M in plasma in patients treated with A (A group) as a first-line EGFR-TKI was lower than with G/E followed by A (G/E→A group), although the differences were not significant (EGFR-driver: 41% [A] vs. 67% [G/E→A], *P*=0.1867; and T790M: 8% [A] vs. 17% [G/E→A], *P*=0.5798). In first-line setting, the detection rate for EGFR-driver and T790M in plasma by cobas was lower in A group than in G/E group, although there was no significant difference (EGFR-driver: 34% [A] vs. 52% [G/E], *P*=0.2072; and T790M: 10% [A] vs. 27% [G/E], *P*=0.1161).

**Conclusion:**

The detection of EGFR-driver and T790M in plasma by cobas in patients treated with afatinib might be lower than with G/E in a real-world setting.

**Supplementary Information:**

The online version contains supplementary material available at 10.1186/s12885-020-07777-2.

## Background

Epidermal growth factor receptor (EGFR) tyrosine kinase inhibitors (TKIs) has prolonged progression-free survival (PFS) compared with platinum-doublet chemotherapy as initial systemic therapy, and have been a standard first-line therapy for patients with *EGFR* mutations [1–3]. First-generation EGFR-TKIs, gefitinib and erlotinib, reversibly bind to and inhibit EGFR signaling, while second-generation EGFR-TKIs, such as afatinib and dacomitinib, irreversibly block the signaling from all relevant homo-dimers and hetero-dimers of the ErbB family receptors. Second-generation EGFR-TKIs have been reported to show a significantly longer PFS than first-generation EGFR-TKIs [4, 5].

The emergence of the EGFR T790M point mutation is the most common mechanism of acquired resistance to the EGFR-TKIs gefitinib, erlotinib and afatinib [6, 7]. Osimertinib, a third-generation and irreversible mutant-selective EGFR-TKI, has been approved for advanced NSCLC patients harboring *EGFR* mutations, including the T790M mutation, based on the results of the AURA3 trial [8]. On the other hand, EGFR wild-type amplification has also been reported as a mechanism or resistance to EGFR-TKIs, including osimertinib [9, 10].

Cell-free DNA (cfDNA) genotyping in plasma using the cobas EGFR Mutation Test v2 (cobas test) is the first liquid biopsy to be approved as a companion diagnostic test to identify patients with the EGFR T790M mutation. cfDNA genotyping in plasma is a more easily accessible method of detecting T790M mutation than tissue-based biopsies. However, the AURA3 trial observed that only 51.2% of T790M-positive patients as evaluated using tumor tissues had T790M mutation as assessed using cfDNA in plasma [8], implying that the sensitivity of the cfDNA assay was insufficient to identify all T790M mutant-positive patients. Nevertheless, few reports have investigated the clinical utility of a liquid biopsy for detecting T790M mutation in patients with acquired resistance to afatinib, since most patients enrolled in the AURA3 trial were treated with gefitinib or erlotinib.

Therefore, we planned to investigate the clinical utility of a liquid biopsy for detecting T790M mutation in EGFR-mutated NSCLC patients with acquired resistance to afatinib. In addition, we evaluated the difference in the detection of T790M in plasma from patients treated with first-generation EGFR-TKIs, and an EGFR wild-type amplification status in patients with acquired resistance to afatinib.

## Methods

### Patients

**Fig. 1:**
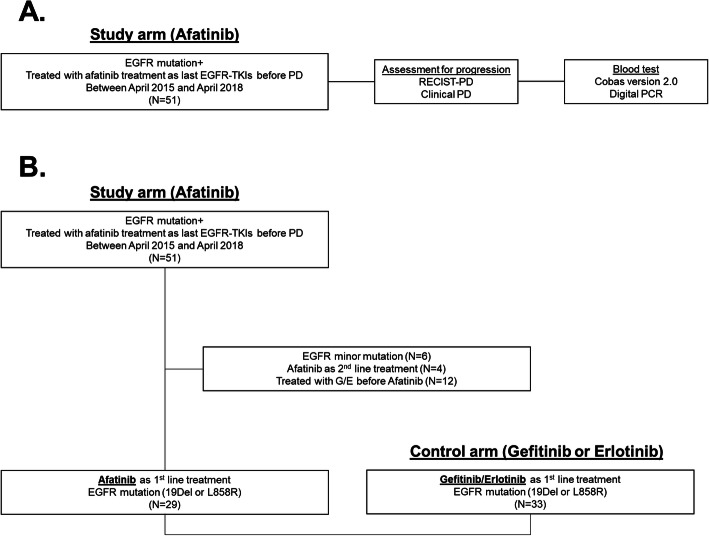
Study schema. **a** A prospective observational study in which plasma samples were collected from patients with acquired resistance to afatinib (*N*=51). **b** A comparison study of detection of EGFR driver and T790M mutation in plasm as assessed using the cobas test in between patients treated with afatinib as first-line setting enrolled in a prospective observation study and patients treated with G/E as first-line setting in a retrospective study (*N*=62)

We studied two patient populations: a study arm consisting of patients enrolled in a prospective observational study, and a control arm consisting of patients in a retrospective study. For the study arm, we prospectively collected plasma samples from 51 patients who had been treated with afatinib, and had experienced progression during afatinib treatment between April 2015 and November 2016 at 13 institutions (Fig. 1a). The inclusion criteria were as follows: 1) a diagnosis of NSCLC, 2) a diagnosis of EGFR mutation, 3) the presence of progressive disease (PD) as assessed using the RECIST criteria, and 4) treatment with afatinib as the last EGFR-TKI to be administered prior to PD. Patients who had been treated with gefitinib/erlotinib (G/E) as the last EGFR-TKIs before RECIST-PD were excluded. The presence of EGFR driver and/or T790M mutation was evaluated using the cobas test and digital droplet PCR (ddPCR).

In addition, to evaluate the difference in the detection of EGFR-driver and T790M mutation in plasma from patients treated with first-generation EGFR-TKIs (G/E) and afatinib as initia EGFR-TKIs, we retrospectively collected data on 33 patients who had been treated with G/E as first-line EGFR-TKIs setting, and whose plasma samples had been analyzed using the cobas test to evaluate the presence of T790M mutation; these patients were regarded as a control arm (Fig. 1b). Moreover, if sufficient cfDNA samples were available after cobas testing and ddPCR for the T790M mutation, EGFR copy number variation (CNV) was evaluated in an exploratory manner using ddPCR.

In all the patients, the patient characteristics, efficacy of EGFR-TKIs, and the T790M status in plasma, objective response rate (ORR), and PFS after treatment with EGFR-TKIs were reviewed. The protocol for this study was approved by the institutional review board of each institution, and the study was registered as a clinical trial (Clinical trial information: UMIN000025112).

### EGFR mutation and wild-type CNV assay in plasma

To assess the presence of EGFR mutation (EGFR driver and T790M mutation) in plasma, approximately 20 mL of whole blood was collected using K2-EDTA collection tubes; within 4 h after blood collection, the samples were then centrifuged to separate the plasma from the peripheral blood cells at 1500 ×*g* for 10 min at 4 °C, and the plasma supernatant was transferred to conical tubes and stored at − 80 °C until transport. The plasma samples were transported at − 80 °C to one of two *commercial* laboratories (SRL Inc., Tokyo, Japan, and G&G Science Inc., Fukushima, Japan). The cobas EGFR Mutation Test v2 (Roche Molecular Systems, CA, USA) was used to detect EGFR mutations in the extracted plasma cfDNA. The mutant allele frequency and EGFR copy number were measured using the QX200 Droplet Digital PCR System (Bio-Rad, Hercules, CA). Isolated cfDNA was amplified using ddPCR Supermix for Probes (Bio-Rad) using EGFR T790M/L858R (PrimePCR ddPCR Mutation Assay; Bio-Rad), EGFR del19 assay (TaqMan Mutation Detection Assay; Thermo Fisher Scientific) for del19 EGFR mutations, and EGFR (RPP30 reference gene) for gene CNV (PrimePCR ddPCR Copy Number Assay; Bio-Rad), according to the manufacturers’ protocols. The ddPCR data were analyzed using Quanta Soft analytical software (version 1.7.4, Bio-Rad).

### Statistical analysis

All the statistical analyses were performed using the JMP version 11 statistical software package (SAS Institute, Cary, NC, USA). Differences in the baseline characteristics between the groups were compared using the Fisher exact tests for categorical data. The response of treatment was evaluated based on Response Evaluation Criteria in Solid Tumors (RECIST version 1.1) [11]. The PFS was calculated from the date of therapy initiation to disease progression. The survival probabilities were estimated using the Kaplan–Meier method, where differences in the variables were calculated using the log-rank test. The cut-off date was August 30, 2018.

## Results

### Patient characteristics

Between April 2015 and November 2016, a total of 51 patients were enrolled in a prospective observational study (Fig. 1a). The characteristics of the patients are summarized in Table 1. The median age of the patients was 67 (range, 36–81) years. Twenty-eight (55%) were male. All the patients had adenocarcinoma, although the selection criteria allowed other NSCLC histology than adenocarcinoma. Forty-five (88%) patients had an ECOG PS score of 0 or 1, while 6 (12%) patients had an ECOG PS or 2 or 3. Thirty-two (63%) had a 19del mutation, 13 (25%) had L858R, and 6 (12%) had minor EGFR mutations. Thirty-nine (76%) patients received treatment with afatinib as a first-line EGFR-TKI setting (A group), and 12 (26%) patients received afatinib as a second or subsequent EGFR-TKI therapy after gefitinib and/or erlotinib (G/E→A group).

**Table 1:** Patient characteristics in a prospective observational study (*N* = 51)

Characteristics	***N*** = 51	%
Age, median (range)	68 (36–81)	
Sex (Male/Female)	28/23	55/45
Smoking (Yes/No)	25/26	49/51
EGFR mutation (19del/L858R/others)	32/13/6	63/25/22
Stage at the diagnosis (III/IV/postoperative recurence)	1/45/5	2/88/10
Performance status		
0–1/2	45/6	88/12
Treatment line with afatinib		
First-line	39	76
Second or subsequent	12	24

### Detection of EGFR driver and T790M mutation in plasma in patients treated with afatinib

**Fig. 2:**
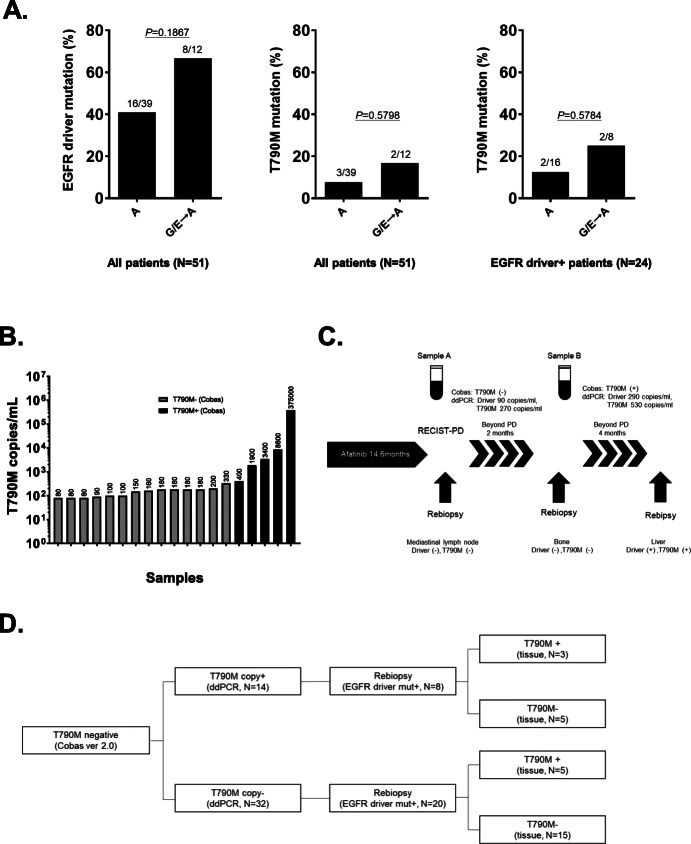
Results of the prospective observational study. **a** Frequency of EGFR driver (left), T790M mutation in all patients (middle) and T790M mutation in EGFR driver mutation positive patients in plasma (right) according to the treatment line of afatinib (first-line vs. second or subsequent EGFR-TKIs setting after gefitinib and/or erlotinib). **b** T790M mutation copy numbers in plasma as assessed using ddPCR. **c** Serial analysis for T790M mutation in plasma obtained from a patient who continued afatinib treatment after RECIST-PD. **d** T790M mutation status in plasma as assessed using ddPCR and in tissue samples in patients with T790M negative results using the cobas test

Among 51 patients, 24 (47%) and 5 (9.8%) patients were positive for EGFR driver and T790M in plasma as assessed using the cobas test, respectively. In the A group (39 patients), 16 (41%) patients were positive for a driver mutation and 3 (8%) patients were positive for T790M in plasma using the cobas test at the time of progression. In the G/E→A group (12 patients), 8 patients (67%) were positive for a driver mutation and 2 (17%) patients were positive for T790M in plasma. The detection rates for EGFR driver and T790M mutations in plasma in the A group were lower than the G/E→A group, although the differences were not significant (Fig. 2a). Regarding the results of ddPCR, 19 (37%) of the 51 patients had T790M mutation copies in plasma using ddPCR, and the T790M copy number ranged from 80 to 375,000 (Fig. 2b). Among these patients, 5 patients who had more than 400 copies/mL also tested positive for the T790M mutation using the cobas test. In one patient who continued to receive afatinib treatment after RECIST-PD, blood samples were collected serially (Fig. 2c). The result of the first cfDNA analysis for T790M mutation using the cobas test was negative. However, the copy numbers for EGFR driver mutation and T790M increased as the site of metastasis progressed, and the second cfDNA analysis using cobas test resulted in a positive result for T790M mutation.

Regarding the concordance between the results of T790M mutation from plasma and tissue samples in patients with T790M mutation negative in plasma as assessed using the cobas test, 8 of the 14 patients with T790M copy positive in plasma using ddPCR underwent rebiopsy, and 3 of these patients had T790M mutation in tissue (Fig. 2d). In contrast, 20 of the 32 patients with T790M copy negative using ddPCR underwent rebiopsy, and 5 patients had T790M mutation in tissue. The presence of T790M mutation copy as detected using ddPCR did not influence the T790M mutation status in tissue obtained from patients with T790M negative results in plasma using the cobas test (37.5% vs. 40%, *P*=0.6508). In addition, we evaluated the PFS of initial-TKIs according to T790M mutation positivity (T790M copy: ≥400 copies, 0< T790M copy: < 400, and T790M copy: negative). The copy number of T790M mutation as assessed using ddPCR did not affect the PFS (Supplemental Fig. 1).

### Detection rate of EGFR driver and T790M mutation between Gefitinib/Erlotinib and Afatinib groups

Next, we evaluated whether the types of EGFR-TKIs (G/E or afatinib) as first-line EGFR-TKI affected the detection of EGFR driver and T790M mutations in plasma. Among the 51 patients who were enrolled in the prospective observational study, 29 patients who were treated with afatinib in a first-line setting and who had major EGFR mutations (Ex19 del and L858R) were selected (A arm). In addition, we retrospectively collected data from 33 patients who had been treated with only first-generation EGFR-TKIs (G/E) as a first-line setting and whose plasma samples had been assessed using the cobas test (G/E arm). The patient characteristics are shown in Table 2. The prevalences of males, smokers, and EGFR 19del were significantly higher in the A arm than in the G/E arm (males: 68.9% vs. 27.2%, *P*< 0.01; smoking: 44.8% vs. 24.2%, *P*=0.01; EGFR 19del: 79.3% vs. 51.5%, *P*=0.01).

**Table 2:** Patient Characteristics in the comparison study (N=62)

	Afatinib (***N***=29).	G/E (***N***=33).	***P*** value
Age, median (range)	66 (36–80)	68 (41–80)	
Sex (Male/Female)	20/9	9/24	0.0010
Histology (Adenocarcinoma)	29	33	1.0
Smoking (Yes/No)	13/16	8/25	0.0126
Mutation (19del/ L858R)	23/6	17/16	0.0225
Stage (III,IV/postoperative recurrence)	25/4	23/10	0.1208
Treatment with Osimertinib (Yes/No)	9/20	15/18	0.2448

*G/E* gefitinib/erlotinib

**Fig. 3:**
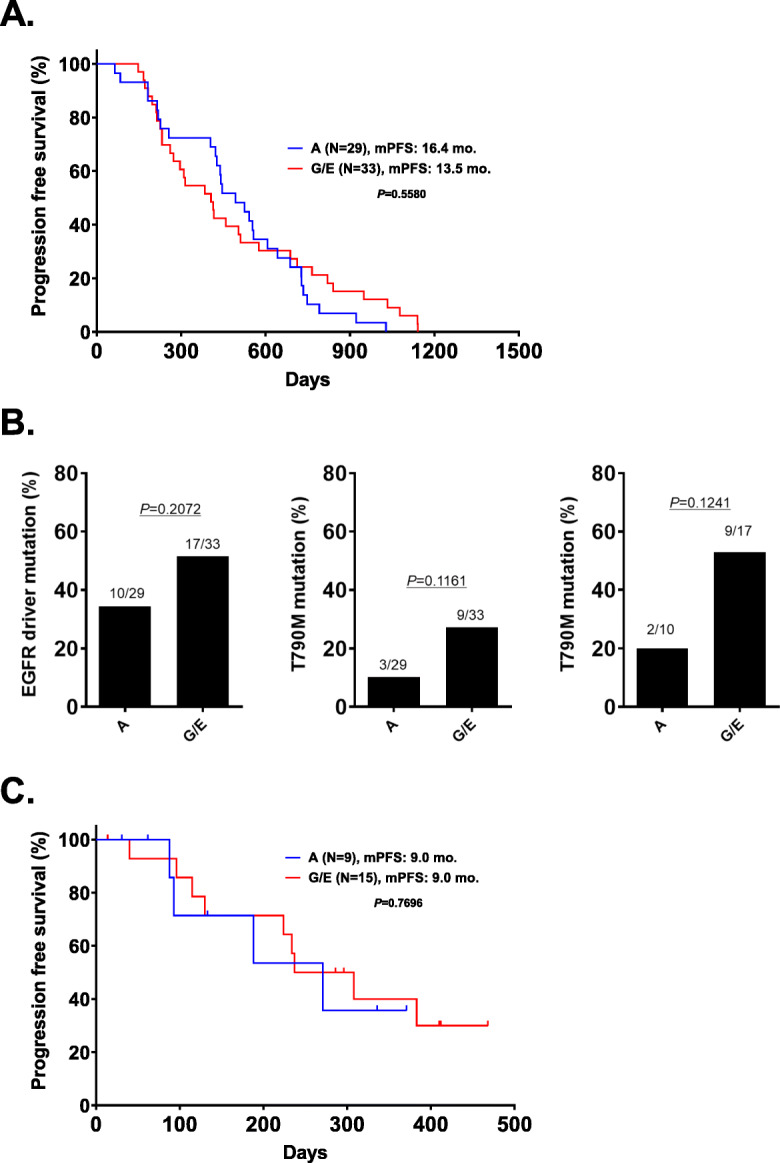
Results of the comparison study. **a** PFS in patients treated with G/E or afatinib in a first-line EGFR-TKI setting. **b** Frequency of EGFR driver (left), T790M mutation in all patients (middle) and T790M mutation in EGFR driver mutation positive patients in plasma (right) according to the types of EGFR-TKIs (afatinib vs. gefitinib and/or erlotinib). **c** PFS of osimertinib after afatinib and G/E failure

Regarding the efficacies of EGFR-TKIs, no significant difference in PFS was observed between the A arm and the G/E arm (16.4 vs. 13.5 months, *P*=0.5580) (Fig. 3a). Regarding the presence of EGFR-driver and T790M mutations in plasma, the detection rates for EGFR-driver and T790M mutation in plasma using the cobas test in patients treated with afatinib were lower than in those treated with G/E, although the differences were not significant (EGFR driver mutation: 34% [A arm] vs. 52% [G/E arm], *P*=0.2072; and T790M: 10% [A arm] vs. 27% [G/E arm], *P*=0.1161) (Fig. 3b). In addition, among patients who tested positive for the EGFR driver mutation in plasma, the detection rate for T790M mutation was not significant between the G/E and A arms (20% vs. 52.9%, *P*=0.1241).

After initial EGFR-TKIs failure, 9 (31%) patients in the A group and 15 (45%) patients in the G/E group received osimertinib treatment. No significant difference in the PFS after osimertinib treatment was seen between the patients in the A and G/E groups (9.0 months [G/E] vs. 9.0 months [A], *P*=0.7696, Fig. 3c).

### Assessment of EGFR wild-type CNV in plasma

**Fig. 4:**
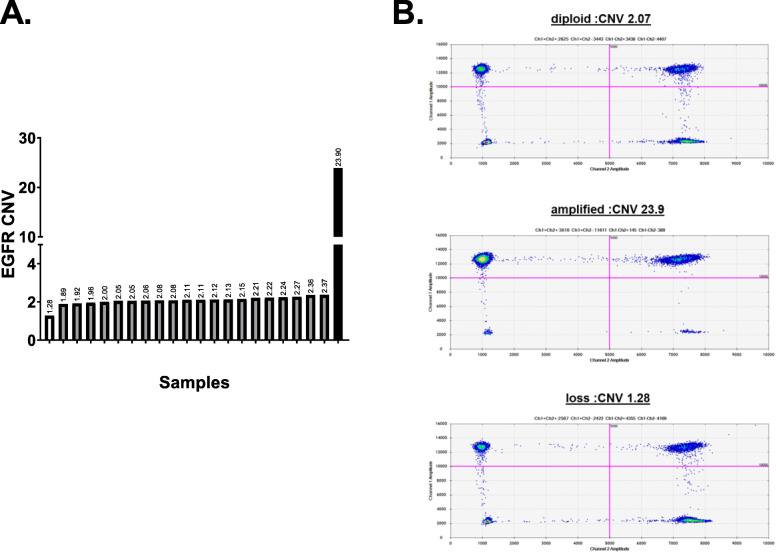
**a** EGFR wild-type copy number variation (CNV) in plasma for EGFR amplification testing. **b** Representative ddPCR data for EGFR CNV in patients with EGFR copy loss, diploid and amplification

In this study, we also analyzed the EGFR wild-type (wt) CNV in cfDNA in an exploratory manner using digital PCR. Twenty-three patients had a sufficient cfDNA volume for the assessment of EGFR-wt copy number. Two of the 23 patients had the T790M mutation in plasma as detected using the cobas test. An EGFR-wt copy number gain and loss were detected in each one patient (EGFR CNV: 1.28 and 23.9) (Fig. 4a and b). Interestingly, the patient who had a high EGFR CNV were treated with osimertinib after afatinib failure, but did not respond to the osimertinib treatment.

## Discussion

In this study, we examined the detection rates for EGFR-driver and T790M mutations in plasma using the cobas test in EGFR-mutated NSCLC patients who had acquired resistance to afatinib in a real-world setting. The detection of T790M mutation in plasma using the cobas test was in complete agreement with the positive results (more than 400 copies/mL) for T790M mutation as assessed using ddPCR. In addition, this study also showed that the detection of EGFR driver and T790M mutations in plasma was lower in patients treated with first-line afatinib than in those with first-line G/E (34.5% vs. 51.5%), although the difference was not significant.

Some reports have shown that in 40–60% of patients with acquired resistance to afatinib, the emergence of T790M mutation was detected using tissue analyses, similar to the results for patients with acquired resistance to G/E [12, 13]. However, Lee et al. recently reported that the occurrence of T790 M mutation in patients treated with afatinib is significantly lower than those with gefitinib and erlotinib [14]. In addition, Yoon et al. reported that although no significant difference was observed, the cumulative acquisition of the T790M mutation in patients treated with afatinib was lower than those with gefitinib (48.8% vs. 59.3%, *P*= .317), since afatinib had a greater inhibitory activity, compared with G/E, against *EGFR*-mutant cell lines harboring common mutations (including T790M) in vitro [15]. These results might be consistent with our data. Regarding the T790M status in plasma in patients treated with afatinib, Hochmair et al. reported that the T790M mutation was detected using a liquid biopsy in 47 out of 67 patients who had been treated with afatinib [16]. However, they did not use the cobas test, but rather the ddPCR system, which is a more sensitive assay than the cobas test. In fact, in this study, among the 46 patients who tested negative for the T790M mutation in plasma using the cobas test, T790M mutation copies were detected using ddPCR in 14 patients. Therefore, the frequency of T790M mutation depends on the types of assays for T790M mutation.

The detection rates for EGFR driver and T790M mutations using the cobas test in our study were lower than those reported in the pooled AURA extension and AURA 2 data (EGFR driver mutations: 76–85% vs. 40–51%, T790M mutations: 61% vs. 5.7–27.2%, respectively) [17]. One possible reason is the difference in terms of patient populations. The AURA studies included only patients with measurable disease to enroll the clinical trials. However, our study only included patients who acquired resistance to an initial TKI treatment, either afatinib or G/E. In addition, the positivity of EGFR driver and T790M mutations in plasma is related to the disease burden, and disease burden can affect positivity. Our data might be closer to the real-world setting for EGFR-mutated NSCLC. Additionally, Sacher et al. reported that when no sensitizing mutation is detected in patients with known EGFR-mutant lung cancer and acquired resistance, plasma genotyping for T790M can be uninformative [18]. To analyze the T790M mutation status in plasma more thoroughly, we evaluated the frequency of T790M mutation in EGFR driver mutation-positive NSCLC patients in plasma, but the detection of T790M mutation in EGFR driver mutation-positive patients was similar between the G/E and A arms.

Additionally, this study also showed that among 23 patients who had a sufficient cfDNA volume for assessment of EGFR wt copy number gain, one patient had EGFR wild-type amplification in cfDNA. EGFR wild-type amplification has also been reported as a mechanism of resistance to EGFR-TKIs, including osimertinib [9, 10]. Indeed, the patient with EGFR wild-type amplification exhibited primary resistance to osimertinib.

The present study had several limitations. First, the sample size was relatively small. In addition, some of the patient characteristics, such as sex, smoking history and EGFR mutation type, differed between the A and G/E arms in the comparison study. However, previous reports have shown that these characteristics did not affect the occurrence of T790M mutation after EGFR-TKIs. Second, the timing for re-biopsy using either tissue or liquid samples was not regulated. Furthermore, tissue analyses for EGFR T790M and EGFR amplification were not performed in all the patients at the same time as the plasma test.

## Conclusions

The detection of EGFR-driver and T790M mutations in plasma using the cobas test might be lower in patients treated with afatinib than with gefitinib/erlotinib in a real-world setting. On the other hands, there was no significant differences of the detection of T790M mutations in tissue between two groups, and types of initial EGFR-TKIs could affect the detection of EGFR-driver and T790M mutations in plasma at the progression. Further examination of the clinical utility of liquid biopsy, including the cobas test and ddPCR, for EGFR driver, T790M mutation and EGFR amplification is needed for the introduction of liquid biopsies into clinical practice in advanced EGFR-mutated NSCLC patients.

## Supplementary Information

**Additional file 1: Supplemental Fig. 1.** PFS of initial-TKIs according to T790M mutation positivity as assessed using ddPCR (T790M copy: ≥400 copies vs. T790M copy: 0.01–400 vs. T790M copy: negative).

## Data Availability

The datasets used and/or analyzed during the current study are available from the corresponding author on reasonable request.

## Abbreviations

EGFR: Epidermal growth factor receptor
TKIs: tyrosine kinase inhibitors
NSCLC: non-small cell lung cancer
PFS: progression free survival
cfDNA: cell free DNA
RECIST: response evaluation criteria in solid tumours
ddPCR: Droplet Digital polymerase chain reaction
CNV: copy number variation
ECOG PS: Eastern Cooperative Oncology Group Performance Status
PD: preogressive disease
