# Supplementary material for: Clinical utility of liquid biopsy for EGFR driver, T790M mutation and EGFR amplification in plasma in patients with acquired resistance to afatinib
Source: BMC Cancer. 2021 Jan 12;21:57. doi: 10.1186/s12885-020-07777-2 (PMC7802126; doi:10.1186/s12885-020-07777-2)

Supplemental Figure. 1

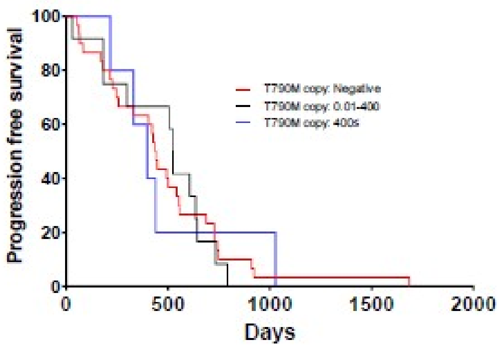

Supplement: Supplementary file 1 [file 12885_2020_7777_MOESM1_ESM.docx]
